# Maximal respiratory pressure after COVID‐19 compared with reference material in healthy adults: A prospective cohort study (The SECURe study)

**DOI:** 10.14814/phy2.16184

**Published:** 2024-09-08

**Authors:** Thora Wesenberg Helt, Jan Christensen, Ronan M. G. Berg, Thomas Kromann Lund, Anna Kalhauge, Frederikke Rönsholt, Daria Podlekareva, Elisabeth Arndal, Flemming Madsen, Mathias Munkholm, Birgitte Hanel, Anne‐Mette Lebech, Terese Lea Katzenstein, Jann Mortensen

**Affiliations:** ^1^ Department of Clinical Physiology and Nuclear Medicine Copenhagen University Hospital–Rigshospitalet Copenhagen Denmark; ^2^ Department of Occupational Therapy and Physiotherapy Copenhagen University Hospital–Rigshospitalet Copenhagen Denmark; ^3^ Department of Biomedical Sciences, Faculty of Health and Medical Sciences University of Copenhagen Copenhagen Denmark; ^4^ Center for Physical Activity Research Copenhagen University Hospital–Rigshospitalet Copenhagen Denmark; ^5^ Department of Cardiology, Section for Lung Transplantation Copenhagen University Hospital–Rigshospitalet Copenhagen Denmark; ^6^ Department of Radiology Copenhagen University Hospital–Rigshospitalet Copenhagen Denmark; ^7^ Department of Infectious Diseases Copenhagen University Hospital–Rigshospitalet Copenhagen Denmark; ^8^ Department of Otorhinolaryngology Copenhagen University Hospital–Rigshospitalet Copenhagen Denmark; ^9^ Center for Clinical Research and Prevention Copenhagen University Hospital–Bispebjerg and Frederiksberg Copenhagen Denmark; ^10^ Department of Clinical Medicine, Faculty of Health and Medical Sciences University of Copenhagen Copenhagen Denmark

**Keywords:** COVID‐19, DALFUMAT, respiratory muscle strength, reference values, SECURe

## Abstract

After COVID‐19 long term respiratory symptoms and reduced lung function including maximal inspiratory pressure (MIP) and maximal expiratory pressure (MEP) have been reported. However, no studies have looked at MIP and MEP in all disease groups and the reference materials collection methods differ substantially. We aimed to determine MIP and MEP in individuals after COVID‐19 infection with different disease severity using reference material of healthy control group obtained using the same standardized method. Patients with COVID‐19 were included March 2020–March 2021 at Rigshospitalet, Denmark. MIP and MEP were measured using microRPM. Predicted MIP and MEP were calculated using reference material obtained from 298 healthy adults aged 18–97 years using the same method. In SECURe, 145 participants were measured median 5 months after COVID‐19 diagnosis and of these 16% had reduced MIP and/or MEP. There was reduced spirometry and total lung capacity, but not reduced diffusion capacity in those with abnormal MIP and/or MEP compared with normal MIP and MEP. Of those with reduced MIP and/or MEP at 5 months, 80% still had reduced MIP and/or MEP at 12 months follow‐up. In conclusion, few have reduced MIP and/or MEP 5 months after COVID‐19 and little improvement was seen over time.

## 1 INTRODUCTION

COVID‐19 has globally infected more than 772 million people with more than 6.9 million deaths as of December 11, 2023 (WHO, 2023). Chest pain, dyspnea and fatigue are some of the symptoms seen to persist in post‐COVID condition (Ballering et al., 2022; Crook et al., 2021). Likewise, low pulmonary diffusing capacity, ventilation‐perfusion defects and lung fibrosis are found in patients after COVID‐19 infection increasing the ventilatory requirements with the most affected being those, who required mechanical ventilation during the infection (Katzenstein et al., 2022). Mechanical ventilation is known to give respiratory muscle weakness and diaphragm dysfunction after as little as 24 h (Levine et al., 2008). Respiratory muscle weakness can cause serious complications including dyspnea, hypoventilation during sleep, atelectasis, and pneumonia and in the most severe cases it can lead to acute respiratory failure (Ricoy et al., 2019).

Diaphragm dysfunction has also been seen in COVID‐19 patients requiring oxygen therapy but not necessarily mechanical ventilation (Boussuges et al., 2022; Farr et al., 2021). Additionally, studies in severe and critical COVID‐19 have shown that patients, who have been on mechanical ventilation had lower maximal expiratory pressure (MEP) and inspiratory pressure (MIP) relative to those who did not (Dosbaba et al., 2023; Krueger et al., 2023; Villelabeitia‐Jaureguizar et al., 2022). Also, disease groups without supplementary oxygen requirement have been shown to have decreased respiratory muscle strength after COVID‐19 (Bostancı et al., 2023; Çelik et al., 2022; Li et al., 2021; Nagel et al., 2022; Plaza & Sevilla, 1992) even though diaphragm dysfunction has not been shown for COVID‐19 infection without oxygen requirement. So, the mechanisms behind the respiratory weakness in COVID‐19 is not known, but other than mechanical ventilation, viral infiltration of the diaphragm, malnutrition, and systemic inflammation which causes catabolism has been also been suggested to play a role (Laghi & Tobin, 2003; Montes‐Ibarra et al., 2022; Shi et al., 2021). Finding the patients who have respiratory muscle weakness after COVID‐19 would make it possible to treat them and hopefully prevent the more severe complications as inspiratory muscle training has been shown to improve diaphragm and inspiratory muscle function and be the best treatment of dyspnea after COVID‐19 (McNarry et al., 2022; Spiesshoefer et al., 2024).

However, none of these studies looking at respiratory muscle weakness after COVID‐19 include all SARS CoV‐2 severity groups, the timing of follow‐up differed, and no standard reference material has been used. Therefore, studies taking these things into account are needed to get an identical assessment across severity groups and an estimation of how many patients are affected by respiratory muscle weakness after COVID‐19. Multiple reference materials for maximal respiratory pressure have been published (Bruschi et al., 1992; Carpenter et al., 1999; Enright et al., 1994, 1995; Evans & Whitelaw, 2009; Gil Obando et al., 2012; Gopalakrishna et al., 2011; Harik‐Khan et al., 1998; Hautmann et al., 2000; Johan et al., 1997; Koulouris et al., 1988; Lavietes et al., 1979; McConnell & Copestake, 1999; McElvaney et al., 1989; Neder et al., 1999; Rodrigues et al., 2017; Vincken et al., 1987; Wilson et al., 1984; Windisch et al., 2004; Wohlgemuth et al., 2003). However, these studies have used different methods of collection; almost all studies are from before 2000 with some of them from before electronic collection was possible; and no study has a reference material for both adults and the elderly (Enright et al., 1994; Evans & Whitelaw, 2009; Windisch et al., 2004). Furthermore, the reference values differ between studies giving different predicted values and degree of changes depending on which reference values were chosen. Therefore, a reference population in both adults and the elderly using the same modern method of collection as in the patients assessed after COVID‐19 is desirable and will ultimately lead to a better determination of changes in respiratory pressure in patients after COVID‐19.

This study aimed to determine maximal respiratory pressure in individuals after COVID‐19 infection with different severity of disease using reference material of healthy control group of both adults and the elderly obtained using the same standardized method. If a reduced value was found 5 months after COVID‐19 infection, reversibility was assessed at 12 months follow‐up.

## 2 MATERIALS AND METHODS

### 2.1 Sequelae of COVID‐19, Copenhagen University Hospital, Rigshospitalet (SECURe)

#### 2.1.1 Study design and population

The SECURe study was a prospective cohort study conducted at Copenhagen University Hospital, Rigshospitalet designed to look at long‐term sequelae of COVID‐19 patients. The inclusion has previously been described (Katzenstein et al., 2022). All patients admitted to the hospital with COVID‐19 from March 2020 to March 2021 were invited to participate in the study. Likewise, nonhospitalized patients tested at the affiliated test site or by word of mouth among health care personnel were invited to participate. COVID‐19 infection was defined as a polymerase chain reaction test verifying SARS‐CoV‐2. Patients were excluded whether they had dementia or were living in an old age facility and being unable to come for follow‐up visits.

#### 2.1.2 Data collection

Lung function testing (dynamic spirometry, body plethysmography, single breath measurement of DLco, MIP, and MEP) 5 months after COVID‐19 infection was performed in accordance with the ERS/ATS guidelines (Macintyre et al., 2005; Miller et al., 2005; Wanger et al., 2005). Patients with MIP and/or MEP values lower than normal were invited to have repeated testing at 12 months postinfection. The lung function variables comprised forced expiratory volume in the first second (FEV1), forced vital capacity (FVC), FEV1/FVC ratio, total lung capacity (TLC), residual volume (RV), RV/TLC ratio and hemoglobin corrected DLco. Age 5 months after COVID‐19 was calculated from the difference between visit date and birth; severity status was grouped as asymptomatic, mild, moderate, severe, and critical according to NIH guidelines and Gandhi et al. 2020; Panel, 2022.

#### 2.1.3 Data handling and statistical analysis

All data were entered into REDcap (13.7.14 © 2023 Vanderbilt University, TN, USA). Analyses were conducted using STATA 18 (StataCorp., Stata Statistical Software: College Station, TX, USA: StataCorp LLC). Percentage of predicted and *z*‐score for MIP and MEP were calculated using formulas derived from the reference material below. MIP and MEP including % predicted and z‐score at five and 12 months after COVID‐19, age, sex and lung function were summarized as percentage (*n*), mean (SD) for normally distributed variables, or median (IQR) for non‐normally distributed variables based on visual inspection of histograms and probability plots. MIP and MEP were defined as normal if *z*‐score >−1.65. Difference between severity groups were assessed using Chi squared for dichotomous data, one‐way ANOVA for normally distributed values and Kruskal–Wallis *H* test for non‐normally distributed data. Difference between lung function in those with normal MIP and MEP and those with abnormal MIP and/or MEP was assessed using Fisher's exact test or Mann Whitney *U*‐test. Differences between first and second assessment for MIP and MEP including % predicted and *z*‐score and difference between those with impaired and normal MIP and MEP at first assessment were assessed using two‐sided *T*‐test with 95% CI for normally distributed values and two‐sided *T*‐test with bias corrected bootstrap 95% CI (10.000 resamples) for non‐normally distributed.

### 2.2 Reference study

#### 2.2.1 Study design and population

From September 11, 2013 to June 18, 2014 a sample of healthy adults aged 18–97 years were recruited. The participants ≥20 years were randomly invited from the Copenhagen General Population Study, which included more than 100.000 participants randomly selected from the Danish Civil Registration System as previously described (Lawlor et al., 2011; Thomsen et al., 2013). The 18 and 19 year old were randomly selected from the Danish Civil Registration System. Inclusion has previously been described (Munkholm et al., 2018). Subjects were randomized to either get MIP or MEP measured first.

#### 2.2.2 Data collection

Body mass index (BMI) was calculated as weight in kg divided by height in m^2^. Finger reach was measured with the arms spread out parallel to the floor from the tip of the left middle finger to the tip of the right middle finger. It was measured to the nearest 1 mm.

#### 2.2.3 Data handling and statistical analysis

All data were entered into Microsoft Excel (2010 © Microsoft 2023, Redmond, WA, USA). All analyses were conducted using STATA 12 and 18 (StataCorp., Stata Statistical Software: College Station, TX, USA: StataCorp LLC). Age, sex, height, weight, BMI, finger reach, MIP, and MEP were summarized as percentage (*n*) or mean ± SD (range). Univariate linear regression was used to assess the association of age, age squared, height, finger reach, weight, and BMI with MIP and MEP for both male and females. The models were stratified by sex. Variables with *p*‐value below 0.1 were included in stepwise model. Height and finger reach were not included in the same model due to collinearity. Reference equations were established using stepwise model selection in multiple linear regression analysis favoring models with lowest Akaike information criterion. The stepwise regression analyses were performed on the whole dataset. Cases with residuals ≥3 SD above or below were checked for validity, and were found to be correct. The root mean squared error (rMSE) measures the average difference between the actual values and the predicted values. The predicted value ±1.96∙rMSE approximates the 2.5th and 97.5th percentile. Z‐scores were calculated as (actual value‐predicted value)/rMSE. Plots showed associations of MIP and MEP with age, height, BMI, weight, and finger reach stratified by sex.

### 2.3 Respiratory muscle pressure measurements in both cohorts

Maximal respiratory pressure was measured using a factory calibrated MicroRPM™ respiratory pressure meter (cat. No. RPM01, CareFusion, Hoechberg, Germany) at room humidity and temperature. The participants were placed on a chair sitting comfortably with loose clothes and correctly positioned without leaning forward or stretching the neck. A nose clip was put on. To measure MIP the participants expired/blew out until RV without the mouth piece, after which the mouth piece was placed in the mouth and maximal inspiration against resistance in minimum 1 second was done (Micro Direct Inc, 2019). The procedure was repeated a minimum of 3 times with small breaks in between. To measure MEP the participant inhaled to TLC without the mouthpiece. Afterwards it was inserted in the mouth and maximal expiration against resistance for a minimum of 1 second was done (Micro Direct Inc, 2019). The procedure was repeated a minimum of 3 times with small breaks in between. If the participant coughed, did a Valsalva's maneuver or air slipped out the measurement was discarded. The difference between the 3 highest values was not allowed to vary more than 10% compared with the highest value. The highest value was used as result for both MIP and MEP. The values for MIP and MEP were both measured as the average of 1 second. The same standard operating procedure was used in both studies with same level of rigor applied to number of efforts and repeatability. Most of the personal were the same in both studies and they received rigorous training in obtaining correct measurements. JM assured continued quality assessment of the personal, measurement, and the equipment. During the reference study, the measurements were implemented as routine at the department. Weight was measured to the nearest 100 g using an electronic scale (Seca model 8,777,021,094, GMBH, Hamburg, Germany), height was measured to the nearest 1 mm using a stadiometer (Holtain limited, Crymych, UK).

## 3 RESULTS

### 3.1 SECURe

In SECURe, 145 participants had maximal respiratory pressure measured 5 months after COVID‐19 with a median of 168 [139; 199] days after COVID‐19 diagnosis and of these (23/145) (Windisch et al., 2004) had reduced MIP and/or MEP. Those with reduced MIP and/or MEP at 5 months were retested at 12 months with a median follow‐up time of 380 [367; 410] days. Across COVID‐19 severity groups, higher age (*p* < 0.001) and higher prevalence of males (*p* < 0.001) were seen in more severe disease (Table 1). No difference was seen in MIP or MEP across COVID‐19 severity groups when taking the predicted value into account (Table 1). Abnormal MIP and/or MEP were detected among individuals with mild (8/23) (Boussuges et al., 2022), severe (12/23) (Bostancı et al., 2023) and critical (3/23) (Ballering et al., 2022) COVID‐19 which was in line with how many were included in each severity group (*p* = 0.20).

**TABLE 1:** Maximal inspiratory and expiratory respiratory pressure 5 months after COVID‐19 for each severity group.

	All (*n* = 145)	Asymptomatic (*n* = 1)	Mild (*n* = 37)	Moderate (*n* = 23)	Severe (*n* = 63)	Critical (*n* = 21)	*P*‐value (between groups)
MIP cmH_2_0	84 [66; 106]	91	82[66; 108]	80 [59; 100]	80 [67; 105]	94 [76; 111]	0.59
MIP %P	95.6 (26.2)	104.6	92.7 (24.5)	92.4 (26.7)	90.8 (28.4)	97.4 (23.4)	0.88
MIP, *z*‐score	−0.29 (0.97)	0.18	−0.28 (0.94)	−0.23 (0.96)	−0.38 (1.04)	−0.14 (0.86)	0.85
MEP cmH_2_0	108 [92; 129]	108	103 [88; 125]	101 [83; 114]	108 [93; 133]	115 [97; 134]	0.48
MEP %P	83.0 (20.2)	88.9	83.2 (18.4)	82.0 (17.0)	82.9 (23.3)	83.6 (18.0)	0.99
MEP, *z*‐score	−0.74 (0.87)	−0.50	−0.75 (0.83)	−0.72 (0.67)	−0.74 (1.01)	−0.73 (0.79)	0.99
Males, *n* (%)	79 (54%)	0 (0%)	12 (32%)	8 (35%)	44 (70%)	15 (71%)	<0.001
Age	54.2 (15.4)	43	44.9 (12.6)	48.8 (19.0)	60.2 (13.2)	59.0 (12.0)	<0.001
Lung function							
FEV1 %P	108.7 [96.0; 121.3]	103.1	111.0 [101.0; 119.2]	108.7 [94.5; 121.7]	111.4 [93.4; 125.0]	97.9 [90.6; 119.2]	0.69
FVC %P	114.0 [98.1; 124.0]	125.8	120.3 [109.6; 127.4]	114.0 [97.2; 124.0]	114.3 [95.9; 121.8]	103.1 [91.4; 119.5]	0.046
FEV/FVC	80.1 [74.8; 83.0]	70.7	79.3 [73.6; 82.7]	81.0 [76.7; 84.1]	80.7 [75.6; 83.1]	80.6 [77.6; 83.6]	0.44
TLC %P	101.8 [90.8; 110.3]	117	109.9 [101.4; 116.3]	106.8 [91.1; 110.3]	100.0 [90.8; 107.2]	86.9 [82.1; 101.8]	<0.001
RV/TLC %P	83.5 [75.1; 92.3]	90.2	83.4 [75.6; 94.4]	85.7 [78.8; 89.7]	83.4 [77.9; 98.0]	72.8 [70.2; 84.7]	0.052
DLco %P	78.7 [71.6; 89.4]	76.7	83.3 [77.6; 99.4]	85.1 [73.6; 94.0]	78.2 [70.8; 85.2]	66.1 [56.8; 76.4]	<0.001

*Note*: *N* (%), Mean (SD), Median [IQR].

Abbreviations: %P, percentage of predicted; FEV1, forced expiratory volume in the first second; FVC, forced vital capacity; MEP, maximal expiratory pressure; MIP, maximal inspiratory pressure; RV, residual volume; TLC, total lung capacity.

For the 23 subjects with reduced MIP and/or MEP at 5 months, (12/23) (Bostancı et al., 2023) had dyspnea. Of the (20/23) (McNarry et al., 2022) were followed‐up at 12 months with no overall improvement in MIP (*p* = 0.14) and in MEP (*p* = 0.52) (Table 2). However, (4/20) (Katzenstein et al., 2022) had normal MIP and MEP at 12 months. Of the three, who only had abnormal MIP at 5 months, all three continued to have abnormal MIP at 12 months whereas one developed abnormal MEP at 12 months. Of the 10 with only abnormal MEP at 5 months, six continued to have abnormal MEP and two developed abnormal MIP (Table 3). For those with both abnormal MIP and MEP, one had normal MIP at 12 months and two had normal MEP at 12 months (Table 3). The subjects with abnormal MIP and/or MEP had similar demographics, anthropometry, symptoms, disease, medication, inflammation, and hospitalization compared with subjects with normal MIP and MEP (Table 4). However, lung function at 5 months was lower among subjects with abnormal MIP and/or MEP compared with subjects with normal MIP and MEP with reduced FEV1%predicted (101.1 [82.0; 111.4] vs. 111.1 [97.2; 123.4], *p* = 0.004), FVC %predicted (102.8 [82.8; 114.8] vs. 115.5 [101.1; 125.6], *p* = 0.002), and TLC %predicted (96.1 [83.9; 101.4] vs. 103.8 [91.1; 111.3], *p* = 0.018), but not DLco %predicted (*p* = 0.85) (Table 4).

**TABLE 2:** Difference in maximal inspiratory and expiratory respiratory pressure between 5 months and 12 months after COVID‐19 for those (*n* = 20) with abnormal maximal inspiratory or expiratory respiratory pressure at 5 months and follow‐up at 12 months.

	5 months	12 months	Difference in mean (95% CI)	*p*‐value
MIP cmH_2_0	68 [47; 74]	60 [39; 74]	−4.05 (−8.72; 1.52)	0.14
MIP %P	60.9 (16.9)	56.4 (21.3)	−4.49 (−9.88; 0.90)	0.097
MIP, *z*‐score	−1.54 (0.69)	−1.71 (0.82)	0.16 (−0.36; 0.04)	0.11
MEP cmH_2_0	83 [59; 95]	84 [70; 95]	2.95 (−4.55; 14.04)	0.52
MEP %P	54.5 (13.6)	56.0 (17.2)	1.57 (−5.42; 8.57)	0.64
MEP, *z*‐score	−2.03 (0.59)	−1.95 (0.74)	0.08 (−0.22; 0.38)	0.61

Note: *N* (%), mean (SD), median [IQR].

Abbreviations: %P, percentage of predicted; MEP, maximal expiratory pressure; MIP, maximal inspiratory pressure.

**TABLE 3:** Classification of abnormal maximal inspiratory and/or expiratory respiratory pressure at 5 months and 12 months after COVID‐19 for those with abnormal maximal inspiratory or expiratory respiratory pressure at 5 months and follow‐up at 12 months (*n* = 20).

	MIP at 12 months		MEP at 12 months	
	Normal	Abnormal	Normal	Abnormal
Only MIP reduced at 5 months (*n* = 3)	‐	3	2	1
Only MEP reduced at 5 months (*n* = 10)	8	2	4	6
Both MIP and MEP reduced at 5 months (*n* = 7)	1	6	2	5

Abbreviations: MEP = maximal expiratory pressure, MIP = maximal inspiratory pressure.

**TABLE 4:** Demographics, anthropometry, hospitalization, symptoms, inflammation, and lung function in those with normal MIP and MEP compared with abnormal MIP and/or MEP at 5 months after COVID‐19.

	Normal MIP and MEP (*n* = 122)	Reduced MIP and/or MEP (*n* = 23)	*p*‐value
Sex (males)	63 (52%)	15 (65%)	0.19
Age (years)	55 [43; 65]	47 [40; 63]	0.19
BMI (kg/m^2^)	27 [24; 31]	29 [25; 34]	0.23
Weight (kg)	81.7 [71.0; 95.3]	87.8 [71.7; 111.0]	0.17
Height (cm)	171.6 [164.4; 178.5]	173.8 [163.7; 183.0]	0.61
Symptoms and disease			
Lung disease	23 (19%)	7 (30%)	0.16
CAT score	6 [2; 10]	5 [2; 11]	0.70
Dyspnea	52 (43%)	12 (52%)	0.20
Hospitalization			
Severity group	Severe [Moderate; Severe]	Severe [Mild; Severe]	0.12
Hospitalization	98 (80%)	18 (78%)	0.51
Length of stay, days	6 [4; 14]	10 [3; 17]	0.28
Mechanical ventilation	15 (14%)	4 (19%)	0.56
Medication			
Remdesivir	34 (30%)	8 (38%)	0.30
Dexamethasone	37 (32%)	10 (48%)	0.14
Antibiotics	53 (46%)	9 (43%)	0.47
Antithrombotic	70 (57%)	15 (65%)	0.86
Inflammation at diagnosis			
Leukocytes (10^9^/L)	5.4 [4.4; 7.4]	6.9 [5.2; 7.8]	0.16
C‐reactive protein (mg/L)	62 [24; 117]	64 [22; 130]	0.97
Lung function			
FEV1 %P	111.1 [97.2; 123.4]	101.1 [82; 111.4]	0.004
FVC %P	115.5 [101.1; 125.6]	102.8 [82.8; 114.8]	0.002
FEV1/FVC %	80.1 [74.8; 82.8]	80.0 [74.2; 84.3]	0.39
TLC %P	103.8 [91.1; 111.3]	96.1 [83.9; 101.4]	0.018
RV/TLC %P	83.3 [74.9; 90.6]	88.0 [78.0; 96.0]	0.31
DLco %P	78.3 [70.9; 90.1]	79.5 [75.5; 84.9]	0.85

*Note*: N (%) or median [IQR].

Abbreviations: %P, percentage of predicted; BMI, body mass index; CAT score, chronic obstructive pulmonary disease assessment test; FEV1, forced expiratory volume in the first second; FVC, forced vital capacity; MEP, maximal expiratory pressure; MIP, maximal inspiratory pressure; RV, residual volume; TLC, total lung capacity.

If a lower limit of normal (LLN) of −1.96 *z*‐score was used as cutoff instead 12 had abnormal MIP and/or MEP at 5 months and 11 (92%) were followed‐up at 12 months. Of these, two (18%) had normal MIP and MEP at 12 months. The one who only had abnormal MIP at 5 months continued to have abnormal MIP, but normal MEP at 12 months. Of the six with only reduced MEP at 5 months, three continued to have abnormal MEP and two developed abnormal MIP. For those with both abnormal MIP and MEP all continued to have abnormal MIP and MEP at 12 months. The 12 with abnormal MIP and/or MEP did not differ from those with normal MIP and MEP with regards to sex, age, anthropometry, symptoms and lung disease, hospitalization, treatment, inflammation, and lung function (data not shown).

### 3.2 Reference study

Of the 310 assessed, 298 (96%) had complete data and were included. Baseline characteristics are presented in Table 5. The participants are equally distributed across age (Figure S1).

**TABLE 5:** Characteristics of the reference study population (*n* = 298).

	Female	Male
Subjects, *n*	152 (51%)	146 (49%)
Age, years	54.0 ± 22.7 (18–97)	54.3 ± 21.3 (18–94)
Height, cm	165.4 ± 7.8 (148.8–186)	179.2 ± 8.0 (158.2–197.5)
Weight, kg	67.6 ± 12.7 (45.1–114.1)	81.2 ± 13.9 (53.8–129)
BMI, kg/m^2^	24.7 ± 4.3 (18.1–43.3)	25.2 ± 3.7 (18.0–41.7)
Finger reach, cm	164.9 ± 7.8 (144.8–186.5)	181.8 ± 8.8 (148.9–202.5)
Maximal inspiratory pressure, cm H_2_O (MIP)	77.7 ± 26.9 (8–147)	101.6 ± 32.5 (25–199)
Maximal expiratory pressure, cm H_2_O (MEP)	107.2 ± 32.1 (40–223)	142.6 ± 41.6 (51–300)

*Note*: Variables are presented as *n* (%) or mean ± SD (range).

**FIGURE 1:**
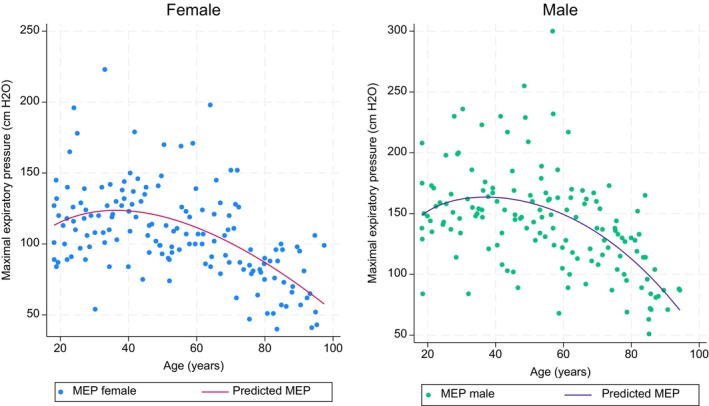
Maximal expiratory pressure depending on age for female and males in the reference study. The line indicates the predicted maximal inspiratory pressure from age using fractional polynomial prediction.

**FIGURE 2:**
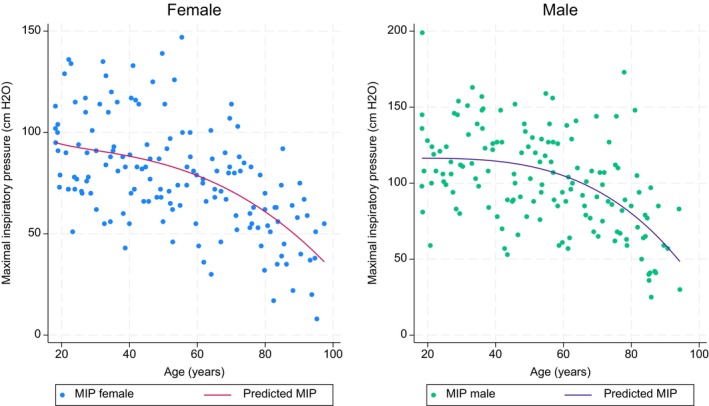
Maximal inspiratory pressure depending on age for female and males in the reference study. The line indicates the predicted maximal expiratory pressure from age using fractional polynomial prediction.

In univariate analyses we found that age, age squared, height, and finger reach were associated with MIP and MEP in both females and males, whereas weight was only associated with MIP and MEP for males (Tables 6 and 7). The final reference equations found using Akaike information criteria were based on age squared and weight for MIP in males, age squared and height for MIP in females. However, for MEP the reference equations were based on age, age squared and weight for males and age and age squared in females (Table 8). The multivariable models for the research equations can be seen in Tables S1 and S2 and the reference equation for MIP in females using finger reach instead of height can be seen in Table S3. For both MIP and MEP there was a large variance in the measurement. However, there was an overall decrease with increasing age in both MIP and MEP for females and males (Figures 1 and 2). The correlation between MIP and MEP and correlations of MIP and MEP with height, weight, BMI, or finger reach can be seen in Figures S2–S10.

**TABLE 6:** Univariate linear regression of age, age^2^, BMI, height, finger reach, and weight as correlates for maximal inspiratory pressure in 298 adults.

	Female			Male		
	B (95% CI)	*p*‐value	*R* ^2^	B (95% CI)	*p*‐value	*R* ^2^
Age (years)	−0.6 (−0.8; −0.5)	<0.001	0.29	−0.7 (−1.0; −0.5)	<0.001	0.22
Age^2^ (years^2^)	−0.006 (−0.007; −0.004)	<0.001	0.31	−0.007 (−0.009; −0.005)	<0.001	0.25
Height (cm)	1.4 (0.9; 2.0)	<0.001	0.17	1.7 (1.1;2.3)	<0.001	0.17
Weight (kg)	0.3 (−0.9; 0.6)	0.14	0.01	0.7 (0.3;1.1)	<0.001	0.09
BMI (kg/m^2^)	−0.7 (−1.7; 0.3)	0.18	0.01	1.0 (−0.5;2.4)	0.19	0.01
Finger reach (cm)	1.2 (0.7; 1.7)	<0.001	0.11	0.9 (0.3; 1.5)	0.002	0.06

**TABLE 7:** Univariate linear regression of age, age^2^, BMI, height, finger reach, and weight as correlates for maximal expiratory pressure in 298 adults.

	Female			Male		
	B (95% CI)	*p*‐value	*R* ^2^	B (95% CI)	*p*‐value	*R* ^2^
Age (years)	−0.7 (−0.9; −0.5)	<0.001	0.24	−0.9 (−1.2; −0.6)	<0.001	0.21
Age^2^ (years^2^)	−0.007 (−0.008; −0.005)	<0.001	0.28	−0.009 (−0.1; −0.007)	<0.001	0.26
Height (cm)	1.4 (0.8;2.1)	<0.001	0.11	2.2 (1.4;2.9)	<0.001	0.16
Weight (kg)	0.3 (−0.07;0.7)	0.11	0.01	0.9 (0.4;1.3)	<0.001	0.08
BMI (kg/m^2^)	−0.4 (−1.6;0.8)	0.48	0.003	1.1 (−0.8;2.9)	0.25	0.002
Finger reach (cm)	1.2 (0.5; 1.8)	<0.001	0.07	1.3 (0.6; 2.1)	0.001	0.07

**TABLE 8:** Reference equations for maximal inspiratory and expiratory pressure.

	Multiple linear regression equation	*R* ^2^	Root mean squared error
Maximal inspiratory pressure			
Male	81.038–0.0065∙age^2^ + 0.5241∙weight	0.30	27.285
Female	−4.8171 ‐0.00496∙age^2^ + 0.6018∙height	0.33	22.045
Maximal expiratory pressure			
Male	87.411 + 1.8660∙age–0.0253∙age^2^ + 0.4912∙weight	0.34	34.318
Female	97.969 + 1.3287∙age–0.0182∙age^2^	0.30	26.831

*Note*: Age in years, height in cm, and weight in kg. Maximal inspiratory and expiratory pressure in cmH_2_O.

If all subjects with BMI >30 were excluded from the study, 267 subjects were included. In univariate analyses we found that age, age squared, height, and finger reach were associated with MIP and MEP in both females and males, whereas weight was associated with MIP for males and MEP for both males and females. BMI was only associated with MIP in males (Tables S4 and S5). The final reference equations found using Akaike information criteria included the same variables as the models with BMI >30 included except for MEP in females where including weight gave a better model. The models can be seen in Table S6.

## 4 DISCUSSION

This is the first study that has looked at changes in maximal respiratory pressure for all severity groups of COVID‐19, performed a follow‐up for a year for those with decreased maximal respiratory pressure, and included a reference material of healthy controls in all age groups using the same method and equipment. Overall, we found that 16% had reduced MIP and/or MEP 5 months after COVID‐19 but of these only 20% had normalized their MIP and/or MEP after 12 months.

### 4.1 SECURe

We found that those with reduced MIP and/or MEP did not differ from those with normal MIP and MEP at 5 months after COVID‐19 except for lung function with lower FEV1 % predicted, FVC % predicted, and TLC %predicted. This reduction in lung function can be due to the respiratory muscle weakness which leads to reduced maximal inspiration or expiration leading to reduced FVC %P and subsequent reduced FEV1 %P. The reduced TLC %P can be explained by the low MIP, whereas we would expect an increase in RV and thus increased RV/TLC %P with a low MEP. However, we did not find an increase in RV/TLC %P which may be because the respiratory muscle weakness is not severe enough or the groups were too small to find the difference. The reduction in lung function disappeared if the cutoff was lowered from LLN of −1.64 to LLN of −1.96, which may be because of the small numbers in the group.

The mechanisms behind the reduced MIP and MEP are not clear. However, mechanical ventilation has been shown to give respiratory muscle weakness and diaphragm dysfunction. This is due to the atrophy and proteolysis of the respiratory muscle because of inactivity while on mechanical ventilation (Levine et al., 2008). However, as only 19% had mechanical ventilation other factors contribute to the abnormal respiratory muscle pressure. Myopathy can also be due to the systemic inflammation in sepsis as there is an increase in cytotoxic nitric oxide and free radicals as well as ubiquitin‐proteasome proteolysis (Laghi & Tobin, 2003). Also, glucocorticoid can contribute to myopathy as they have a catabolic effect (Laghi & Tobin, 2003). Mechanical ventilation, inflammation and glucocorticoid can also give neuropathy and 60%–90% of those who survive intensive care get normal respiratory muscle function and more than one‐third of those with severe involvement still have reduced respiratory muscle function after 2 years (De Jonghe et al., 2002; de Sèze et al., 2000; Leijten et al., 1995). It is more likely to happen if the blood glucose level is not properly regulated (Laghi & Tobin, 2003). It is unknown how the blood glucose regulation was during the hospitalization for the two groups, but 48% got glucocorticoid in the group with abnormal MIP and/or MEP to reduce inflammation, both of which could play a role. Sepsis can also cause mitochondrial dysfunction which can give reduced muscle function (Laghi & Tobin, 2003). This mitochondrial dysfunction is also seen with endotoxemia which has been found in patients with critical COVID‐19 (Khan et al., 2022; Laghi & Tobin, 2003). Viral infiltration of the diaphragm could also contribute as damage to the muscles can cause long‐lasting muscle fatigue (Laghi & Tobin, 2003; Shi et al., 2021). Also, load‐induced injury and hypocapnia can give long‐lasting muscle fatigue, all of which can contribute to the reduction in respiratory muscle pressure, however we have no information on these parameters. So, the mechanism behind reduced respiratory muscle pressure is most likely multifactorial. However, as respiratory muscle weakness can cause serious complications and in the most severe cases it can lead to acute respiratory failure (Ricoy et al., 2019), finding the patients who have respiratory muscle weakness after COVID‐19 is important to prevent the more severe complications. More invasive methods can be used to confirm the respiratory muscle weakness such as diaphragm electromyography and they can subsequently be treated with inspiratory muscle training to improve diaphragm and inspiratory muscle function (McNarry et al., 2022; Spiesshoefer et al., 2024).

We found that overall, both MIP and MEP were within the normal range with MIP of 95.6% predicted and MEP of 83.0% predicted at 5 months after COVID‐19 diagnosis. This differs from other studies where the mean MIP ranged from 40% to 100% predicted with 5 of 7 studies between 70 and 87% predicted (Çelik et al., 2022; Collet et al., 2023; Del Corral et al., 2023; Güneş et al., 2023; Krueger et al., 2023; Nagel et al., 2022; Villelabeitia‐Jaureguizar et al., 2022). Five studies have looked at mean MEP % predicted with a range from 50 to 93, but with two studies reporting below 60% predicted (Çelik et al., 2022; Del Corral et al., 2023; Güneş et al., 2023; Krueger et al., 2023; Villelabeitia‐Jaureguizar et al., 2022). This difference between studies may have different explanations. One of them is the difference in time of measurement after COVID‐19. The studies measured from right at diagnosis (Li et al., 2021), after hospitalization (Villelabeitia‐Jaureguizar et al., 2022), around 3 months after COVID‐19 (Çelik et al., 2022; Krueger et al., 2023; Nagel et al., 2022), at 6 months (Güneş et al., 2023; Plaza & Sevilla, 1992), and up to 1 year after COVID‐19 (Del Corral et al., 2023). Another study had maximal respiratory values measured before COVID‐19 diagnosis and at multiple follow‐up times after. They found that after 52 days the participants were almost back to normal maximal respiratory values after a reduction right after COVID‐19 diagnosis (Bostancı et al., 2023). However, the study participants were professional athletes training 6 times a week, which may speed up recovery. We may find so few with reduced respiratory values because the first measurement was after 5 months and the recovery has happened for most of them. A study looking at MIP at diagnosis found that 20% had an abnormal MIP, this fits with our findings but they used a cutoff of 50% of predicted. This is a lower cutoff than in our study, so the prevalence is most likely higher (Li et al., 2021). This suggests that maximal respiratory pressure is affected at diagnosis, but most patients recover within the first couple of months. Our study did not find a difference between COVID‐19 severity groups. This is supported by Krueger et al. who did not find any difference between groups (Krueger et al., 2023). Li et al. (Li et al., 2021) did, however, find a difference between groups with the highest number of patients with abnormal MIP in those with moderate and severe but not critical disease. Other studies did not include as many disease groups. Nonetheless, abnormal respiratory pressure was reported in both studies with mild disease and studies with critical disease, suggesting that other factors than disease severity are important in development of reduced respiratory pressure.

Another relevant reason for the difference between studies was that some of those with longer follow‐up times were enrolled from either post‐COVID‐19 symptoms clinics or rehabilitation clinics. This means that only participants with persistent symptoms such as dyspnea or need for rehabilitation were included (Collet et al., 2023; Del Corral et al., 2023; Nagel et al., 2022). They are more likely to be affected as dyspnea has been shown to be associated with reduced maximal respiratory strength (Nagel et al., 2022). Meaning they cannot shed light on the nature of recovery of reduced respiratory muscle strength. However, those with mild disease followed‐up at 6 months after COVID‐19 were healthy young participants, who do not necessarily have persistent symptoms (Güneş et al., 2023; Plaza & Sevilla, 1992). For those with symptoms or need of rehabilitation, multiple studies have used respiratory training programs to decrease the symptom burden, improve respiratory muscle strength and quality of life. Training programs were between 6 and 12 weeks and seemed to improve maximal respiratory pressure (Chen et al., 2023; Collet et al., 2023; Del Corral et al., 2023; McNarry et al., 2022; Metcalfe et al., 2023). One study found that the effect of training on dyspnea was associated with increase in MIP and that those starting training before 3 months after COVID‐19, being younger at diagnosis, and having milder dyspnea had better effect of training (Metcalfe et al., 2023). However, a previous study on inspiratory muscle training in healthy adults showed increase in MIP but not in FEV1, FVC, maximal oxygen uptake, peak expiratory flow, and work capacity (Hanel & Secher, 1991). In another study only those who were admitted to intensive care had an increase in respiratory MIP with no difference in the hospitalized non‐intensive care group (Villelabeitia‐Jaureguizar et al., 2022). So decrease in respiratory muscle strength seems to have a large part of reversibility.

Another likely explanation of the difference between studies is that the way of measuring maximal respiratory pressure, the use of reference equations, and the cutoff for when maximal respiratory pressure is abnormal differs between studies. For example, a group of elite volleyball players without COVID‐19 infection had a MEP of 67% of expected (Çelik et al., 2022). This is lower than we would expect for healthy individuals training 6 times a week suggesting that the reference equations are not optimal. Therefore, we included our own reference material making sure the equipment and method were the same in both populations.

### 4.2 Reference study

Multiple reference materials for maximal respiratory pressure have previously been published (Bruschi et al., 1992; Carpenter et al., 1999; Enright et al., 1994, 1995; Evans & Whitelaw, 2009; Gil Obando et al., 2012; Gopalakrishna et al., 2011; Harik‐Khan et al., 1998; Hautmann et al., 2000; Johan et al., 1997; Koulouris et al., 1988; Lavietes et al., 1979; McConnell & Copestake, 1999; McElvaney et al., 1989; Neder et al., 1999; Rodrigues et al., 2017; Vincken et al., 1987; Wilson et al., 1984; Windisch et al., 2004; Wohlgemuth et al., 2003). Most of them use a tube mouthpiece, but some use a flange. Also the gauge type differs as some of the studies are from before there was a possibility of the electronic gauge type and aneroid was used instead (Evans & Whitelaw, 2009). Also, different studies included different variables as predictors in reference equations. We found that height (females), weight (males), and age squared gave the best model for MIP, whereas age, age squared, and weight (males) gave the best model for MEP. However, those models only explained 30%–34% of our variance. Other studies found different relevant variables. Some studies only included age as a linear parameter (Evans & Whitelaw, 2009; McElvaney et al., 1989; Windisch et al., 2004). Yet, on our graphs for MIP and MEP with age, the relationship was not linear. As MIP and MEP more rapidly decrease in the elderly, using a linear relationship will overestimate the predicted value for the elderly. For other parameters, one study found that weight, height, BMI, waist girth and calf girth, education level, health status, physical activity and smoking were associated with MIP (Carpenter et al., 1999). Nevertheless, the study only had subjects between 47 and 68 years which makes it less valid in older and younger ages as MIP is age dependent. Similarly, a study in adults aged 20–90 years found the MIP was associated with weight in males and weight and height in females. Other studies found that MEP and MIP were only associated with age in males, whereas they were correlated with height in women (Wilson et al., 1984) and similarly a study found that height and weight were not correlated with MIP and MEP (Vincken et al., 1987). In adults above 65 years an association between MIP and lean body mass was seen (Enright et al., 1994), whereas another study found that body surface area was a better predictor than other anthropometric parameters (Bruschi et al., 1992). Nonetheless, both variables are not as readily available making it less useful than height and weight. Overall, all studies on reference equations found very different results, still all studies agreed that age and sex were important parameters in prediction of maximal respiratory pressure and most study agreed that some evaluation of anthropometry was important. These differences in findings could be because most studies only included adults or the elderly, and that the few studies with a wide age range had very few in the older group (Enright et al., 1994; Evans & Whitelaw, 2009; Harik‐Khan et al., 1998; Windisch et al., 2004). Also, difference in race could play a role as a Brazilian study found that the use of Caucasian references to underestimate the observed MIP (Johan et al., 1997). Lastly, physical activity has been shown to play a role in MIP and MEP (McConnell & Copestake, 1999; Neder et al., 1999). However, physical activity as a variable can be hard to use, as it can be summarized in many different ways and it is unknown wherever duration, intensity, type or frequency is the most optimal parameter. This study is the first to compare with extensive reference values obtained from a healthy control group using the same method and equipment, but also with a broad age range and even distribution across the age range.

### 4.3 Limitations

Our study had some limitations. Firstly, we only had one participant in the asymptomatic group. As patients were only tested if having COVID‐19 symptoms at diagnosis according to national guidelines, it was harder to include participants in this group. However, looking at studies with both asymptomatic and mild COVID‐19, the degree of reduced MIP and MEP seemed similar. Secondly, some of the participants with reduced MIP and/or MEP dropped out and when only so few have abnormal MIP and/or MEP it would have been relevant to see the change for these. However, only three individuals who were not followed up and they were in three different severity groups. Thirdly, there can be some inter‐visit repeatability for those who were followed up. However, in the reference study a subgroup (*n* = 66) was followed up after a week and the inter‐visit repeatability for MIP was 5% whereas the inter‐visit repeatability was 2% in MEP, both of which are small. Fourthly, respiratory muscle pressure measurements are prone to poor technique, however the personal were trained in performing these measurements with coaching of the subjects and continuously performed these measurements routinely reducing measurements with poor technique. Fifthly, a LLN of −1.64 was used where −1.96 should be used in subjects without symptoms. However, more than half the subjects had dyspnea 5 months after COVID‐19 justifying the use of a LLN of −1.64. Lastly, the reference study only included variables which could explain around 30–34% of the variance, so more explaining variables could potentially make a stronger model, however, the currently included variables are easy to measure and readily available reducing the potential for measurement error and the usefulness of the reference equations. Also, the reference study included healthy subjects with BMI above 30 as a large part of the population are overweight or obese. But such a high BMI is not healthy so the models for BMI ≤30 are included in the supplementary material. However, the new model did not change the prediction substantially.

## 5 CONCLUSION

Relatively few individuals had reduced MIP and/or MEP 5 months after COVID‐19 and there was no apparent relationship to disease severity. Moreover, neither MIP nor MEP improved markedly from 5 to 12‐month follow‐up. Further studies looking at differences between individuals with and without reduced respiratory muscle pressures with a longer follow‐up is needed to understand the mechanistic basis of this reduced maximal respiratory pressure, and to uncover for how long the reduced maximal respiratory pressure persists.

## FUNDING INFORMATION

The authors received no financial support specific for this research activity.

## CONFLICT OF INTEREST STATEMENT

The authors declare no conflicts of interest.

## AUTHOR CONTRIBUTION

Conceived and designed research. Jann Mortensen, Flemming Madsen, Anne‐Mette Lebech, Terese Lea Katzenstein. Performed experiment. Jan Christensen, Frederikke Rönsholt, Daria Podlekareva, Mathias Munkholm, Birgitte Hanel, Anne‐Mette Lebech, Terese Lea Katzenstein, Jann Mortensen. Analyzed data. Thora Wesenberg Helt, Jann Mortensen, Ronan M. G. Berg. Interpreted results of experiments. Thora Wesenberg Helt, Jann Mortensen. Prepared figures. Thora Wesenberg Helt. Drafted manuscript. Thora Wesenberg Helt. Edited and revised manuscript. Thora Wesenberg Helt, Jan Christensen, Ronan M. G. Berg, Thomas Kromann Lund, Anna Kalhauge, Frederikke Rönsholt, Daria Podlekareva, Elisabeth Arndal, Flemming Madsen, Mathias Munkholm, Birgitte Hanel, Anne‐Mette Lebech, Terese Lea Katzenstein, Jann Mortensen. Approved final version of manuscript. Thora Wesenberg Helt, Jan Christensen, Ronan M. G. Berg, Thomas Kromann Lund, Anna Kalhauge, Frederikke Rönsholt, Daria Podlekareva, Elisabeth Arndal, Flemming Madsen, Mathias Munkholm, Birgitte Hanel, Anne‐Mette Lebech, Terese Lea Katzenstein, Jann Mortensen.

## ETHICS STATEMENT

All participants gave written consent. The study was approved by the Danish Medical Research Ethics Committees (H‐20028792) (M 1–10–72‐552‐12).

## Supporting information


Figure S1:

Table S1.

Table S2.

Table S3.

Table S4.

Table S5.

Table S6.

## Data Availability

As a university in a Member State of the European Union, University of Copenhagen is obliged to comply with the provisions of the General Data Protection Regulation. Under Article 9 (2), (j) universities can process sensitive personal data for scientific research purposes. In addition, it is stipulated in Article 9, (4) that Member States may maintain or introduce further conditions, including limitations, with regard to the processing of genetic data, biometric data or data concerning health. The Danish legislation has introduced further conditions in Article 10 of the Danish Act on Data Protection. It is stated in Article 10 of the said Act, that personal research data can be transferred to scientific journals for verification of the research results. However, the Danish Act on Data Protection does not allow for personal data to be made available to others without prior individual approval from the Danish Data Protection Agency.

## References

[phy216184-bib-0001] Ballering, A. V. , van Zon, S. K. R. , Olde Hartman, T. C. , & Rosmalen, J. G. M. (2022). Persistence of somatic symptoms after COVID‐19 in The Netherlands: An observational cohort study. Lancet (London, England), 400, 452–461. 35934007 10.1016/S0140-6736(22)01214-4 PMC9352274

[phy216184-bib-0002] Bostancı, Ö. , Karaduman, E. , Çolak, Y. , Yılmaz, A. K. , Kabadayı, M. , & Bilgiç, S. (2023). Respiratory muscle strength and pulmonary function in unvaccinated athletes before and after COVID‐19 infection: A prospective cohort study. Respiratory Physiology & Neurobiology, 308, 103983. 36343877 10.1016/j.resp.2022.103983 PMC9635222

[phy216184-bib-0003] Boussuges, A. , Habert, P. , Chaumet, G. , Rouibah, R. , Delorme, L. , Menard, A. , Million, M. , Bartoli, A. , Guedj, E. , Gouitaa, M. , Zieleskiewicz, L. , Finance, J. , Coiffard, B. , Delliaux, S. , & Brégeon, F. (2022). Diaphragm dysfunction after severe COVID‐19: An ultrasound study. Frontiers in Medicine, 9, 949281. 36091672 10.3389/fmed.2022.949281 PMC9448976

[phy216184-bib-0004] Bruschi, C. , Cerveri, I. , Zoia, M. C. , Fanfulla, F. , Fiorentini, M. , Casali, L. , Grassi, M. , & Grassi, C. (1992). Reference values of maximal respiratory mouth pressures: A population‐based study. The American Review of Respiratory Disease, 146, 790–793. 1519865 10.1164/ajrccm/146.3.790

[phy216184-bib-0005] Carpenter, M. A. , Tockman, M. S. , Hutchinson, R. G. , Davis, C. E. , & Heiss, G. (1999). Demographic and anthropometric correlates of maximum inspiratory pressure: The atherosclerosis risk in communities study. American Journal of Respiratory and Critical Care Medicine, 159, 415–422. 9927352 10.1164/ajrccm.159.2.9708076

[phy216184-bib-0006] Çelik, Z. , Güzel, N. A. , Kafa, N. , & Köktürk, N. (2022). Respiratory muscle strength in volleyball players suffered from COVID‐19. Irish Journal of Medical Science, 191, 1959–1965. 34748144 10.1007/s11845-021-02849-z PMC8573575

[phy216184-bib-0007] Chen, Y. , Liu, X. , & Tong, Z. (2023). Can inspiratory muscle training benefit patients with COVID‐19? A systematic review and meta‐analysis. Journal of Medical Virology, 95, e28956. 37503550 10.1002/jmv.28956

[phy216184-bib-0008] Collet, R. , van Egmond, M. , van der Schaaf, M. , & Major, M. (2023). Feasibility of inspiratory muscle training for patients with persistent DYSPNOEA after COVID‐19 infection: A pilot study. Journal of Rehabilitation Medicine. Clinical Communications, 6, 6507. 37324934 10.2340/jrmcc.v6.6507 PMC10262391

[phy216184-bib-0009] Crook, H. , Raza, S. , Nowell, J. , Young, M. , & Edison, P. (2021). Long covid‐mechanisms, risk factors, and management. BMJ (Clinical Research Ed), 374, n1648. 10.1136/bmj.n1648 34312178

[phy216184-bib-0010] De Jonghe, B. , Sharshar, T. , Lefaucheur, J. P. , Authier, F. J. , Durand‐Zaleski, I. , Boussarsar, M. , Cerf, C. , Renaud, E. , Mesrati, F. , Carlet, J. , Raphaël, J. C. , Outin, H. , & Bastuji‐Garin, S. (2002). Paresis acquired in the intensive care unit: A prospective multicenter study. JAMA, 288, 2859–2867. 12472328 10.1001/jama.288.22.2859

[phy216184-bib-0011] de Sèze, M. , Petit, H. , Wiart, L. , Cardinaud, J. P. , Gaujard, E. , Joseph, P. A. , Mazaux, J. M. , & Barat, M. (2000). Critical illness polyneuropathy. A 2‐year follow‐up study in 19 severe cases. European Neurology, 43, 61–69. 10686462 10.1159/000008137

[phy216184-bib-0012] Del Corral, T. , Fabero‐Garrido, R. , Plaza‐Manzano, G. , Fernández‐de‐Las‐Peñas, C. , Navarro‐Santana, M. , & López‐de‐Uralde‐Villanueva, I. (2023). Home‐based respiratory muscle training on quality of life and exercise tolerance in long‐term post‐COVID‐19: Randomized controlled trial. Annals of Physical and Rehabilitation Medicine, 66, 101709. 36191860 10.1016/j.rehab.2022.101709 PMC9708524

[phy216184-bib-0013] Dosbaba, F. , Hartman, M. , Batalik, L. , Senkyr, V. , Radkovcova, I. , Richter, S. , Brat, K. , Cahalin, L. P. , & Formiga, M. F. (2023). A temporal examination of inspiratory muscle strength and endurance in hospitalized COVID‐19 patients. Heart & Lung, 60, 95–101. 36934476 10.1016/j.hrtlng.2023.03.007 PMC10008810

[phy216184-bib-0014] Enright, P. L. , Adams, A. B. , Boyle, P. J. , & Sherrill, D. L. (1995). Spirometry and maximal respiratory pressure references from healthy Minnesota 65‐ to 85‐year‐old women and men. Chest, 108, 663–669. 7656613 10.1378/chest.108.3.663

[phy216184-bib-0015] Enright, P. L. , Kronmal, R. A. , Manolio, T. A. , Schenker, M. B. , & Hyatt, R. E. (1994). Respiratory muscle strength in the elderly. Correlates and reference values. Cardiovascular health study research group. American Journal of Respiratory and Critical Care Medicine, 149, 430–438. 8306041 10.1164/ajrccm.149.2.8306041

[phy216184-bib-0016] Evans, J. A. , & Whitelaw, W. A. (2009). The assessment of maximal respiratory mouth pressures in adults. Respiratory Care, 54, 1348–1359. 19796415

[phy216184-bib-0017] Farr, E. , Wolfe, A. R. , Deshmukh, S. , Rydberg, L. , Soriano, R. , Walter, J. M. , Boon, A. J. , Wolfe, L. F. , & Franz, C. K. (2021). Diaphragm dysfunction in severe COVID‐19 as determined by neuromuscular ultrasound. Annals of Clinical Translational Neurology, 8, 1745–1749. 34247452 10.1002/acn3.51416 PMC8351384

[phy216184-bib-0018] Gandhi, R. T. , Lynch, J. B. , & Del Rio, C. (2020). Mild or moderate Covid‐19. The New England Journal of Medicine, 383, 1757–1766. 32329974 10.1056/NEJMcp2009249

[phy216184-bib-0019] Gil Obando, L. M. , López López, A. , & Avila, C. L. (2012). Normal values of the maximal respiratory pressures in healthy people older than 20 years old in the City of Manizales—Colombia. Colombia Medica (Cali, Colombia), 43, 119–125. 24893052 PMC4001942

[phy216184-bib-0020] Gopalakrishna, A. , Vaishali, K. , Prem, V. , & Aaron, P. (2011). Normative values for maximal respiratory pressures in an Indian Mangalore population: A cross‐sectional pilot study. Lung India, 28, 247–252. 22084536 10.4103/0970-2113.85684 PMC3213709

[phy216184-bib-0021] Güneş, M. , Yana, M. , & Güçlü, M. B. (2023). Physical activity levels respiratory and peripheral muscle strength and pulmonary function in young post‐COVID‐19 patients: A cross‐sectional study. Wiener Klinische Wochenschrift, 135, 251–259. 37115337 10.1007/s00508-023-02204-5 PMC10141881

[phy216184-bib-0022] Hanel, B. , & Secher, N. H. (1991). Maximal oxygen uptake and work capacity after inspiratory muscle training: A controlled study. Journal of Sports Sciences, 9, 43–52. 1856912 10.1080/02640419108729854

[phy216184-bib-0023] Harik‐Khan, R. I. , Wise, R. A. , & Fozard, J. L. (1998). Determinants of maximal inspiratory pressure. The Baltimore longitudinal study of aging. American Journal of Respiratory and Critical Care Medicine, 158, 1459–1464. 9817693 10.1164/ajrccm.158.5.9712006

[phy216184-bib-0024] Hautmann, H. , Hefele, S. , Schotten, K. , & Huber, R. M. (2000). Maximal inspiratory mouth pressures (PIMAX) in healthy subjects—what is the lower limit of normal? Respiratory Medicine, 94, 689–693. 10926341 10.1053/rmed.2000.0802

[phy216184-bib-0025] Johan, A. , Chan, C. C. , Chia, H. P. , Chan, O. Y. , & Wang, Y. T. (1997). Maximal respiratory pressures in adult Chinese, Malays and Indians. The European Respiratory Journal, 10, 2825–2828. 9493668 10.1183/09031936.97.10122825

[phy216184-bib-0026] Katzenstein, T. L. , Christensen, J. , Lund, T. K. , Kalhauge, A. , Rönsholt, F. , Podlekareva, D. , Arndal, E. , Berg, R. M. G. , Helt, T. W. , Lebech, A.‐M. , & Mortensen, J. (2022). Relation of pulmonary diffusing capacity decline to HRCT and VQ SPECT/CT findings at early follow‐up after COVID‐19: A prospective cohort study (the SECURe study). Journal of Clinical Medicine, 11(19), 5687. 10.3390/jcm11195687 36233555 PMC9572695

[phy216184-bib-0027] Khan, S. , Bolotova, O. , Sahib, H. , Foster, D. , & Mallipattu, S. K. (2022). Endotoxemia in critically ill patients with COVID‐19. Blood Purification, 51, 513–519. 34515062 10.1159/000518230 PMC8450835

[phy216184-bib-0028] Koulouris, N. , Mulvey, D. A. , Laroche, C. M. , Green, M. , & Moxham, J. (1988). Comparison of two different mouthpieces for the measurement of Pimax and Pemax in normal and weak subjects. The European Respiratory Journal, 1, 863–867. 3229485

[phy216184-bib-0029] Krueger, T. , van den Heuvel, J. , van Kampen‐van den Boogaart, V. , van Zeeland, R. , Mehagnoul‐Schipper, D. J. , Barten, D. G. , Knarren, L. , Maas, A. F. G. , Wyers, C. E. , Gach, D. , Schols, A. , Beijers, R. , van den Bergh, J. P. , & van Osch, F. H. M. (2023). Pulmonary function three to five months after hospital discharge for COVID‐19: A single centre cohort study. Scientific Reports, 13, 681. 36639404 10.1038/s41598-023-27879-8 PMC9839688

[phy216184-bib-0030] Laghi, F. , & Tobin, M. J. (2003). Disorders of the respiratory muscles. American Journal of Respiratory and Critical Care Medicine, 168, 10–48. 12826594 10.1164/rccm.2206020

[phy216184-bib-0031] Lavietes, M. H. , Clifford, E. , Silverstein, D. , Stier, F. , & Reichman, L. B. (1979). Relationship of static respiratory muscle pressure and maximum voluntary ventilation in normal subjects. Respiration, 38, 121–126. 515536 10.1159/000194068

[phy216184-bib-0032] Lawlor, D. A. , Harbord, R. M. , Tybjaerg‐Hansen, A. , Palmer, T. M. , Zacho, J. , Benn, M. , Timpson, N. J. , Smith, G. D. , & Nordestgaard, B. G. (2011). Using genetic loci to understand the relationship between adiposity and psychological distress: A Mendelian randomization study in the Copenhagen general population study of 53,221 adults. Journal of Internal Medicine, 269, 525–537. 21210875 10.1111/j.1365-2796.2011.02343.x

[phy216184-bib-0033] Leijten, F. S. , Harinck‐de Weerd, J. E. , Poortvliet, D. C. , & de Weerd, A. W. (1995). The role of polyneuropathy in motor convalescence after prolonged mechanical ventilation. JAMA, 274, 1221–1225. 7563512

[phy216184-bib-0034] Levine, S. , Nguyen, T. , Taylor, N. , Friscia, M. E. , Budak, M. T. , Rothenberg, P. , Zhu, J. , Sachdeva, R. , Sonnad, S. , Kaiser, L. R. , Rubinstein, N. A. , Powers, S. K. , & Shrager, J. B. (2008). Rapid disuse atrophy of diaphragm fibers in mechanically ventilated humans. The New England Journal of Medicine, 358, 1327–1335. 18367735 10.1056/NEJMoa070447

[phy216184-bib-0035] Li, M. , Zhou, C. , Jiang, J. , You, H. , Liu, C. , Shen, P. , & Feng, Z. (2021). Investigations on the respiratory function in COVID‐19 patients: A prospective cohort study. BioMed Research International, 2021, 9928276. 34963882 10.1155/2021/9928276 PMC8710188

[phy216184-bib-0036] Macintyre, N. , Crapo, R. O. , Viegi, G. , Johnson, D. C. , van der Grinten, C. P. , Brusasco, V. , Burgos, F. , Casaburi, R. , Coates, A. , Enright, P. , Gustafsson, P. , Hankinson, J. , Jensen, R. , McKay, R. , Miller, M. R. , Navajas, D. , Pedersen, O. F. , Pellegrino, R. , & Wanger, J. (2005). Standardisation of the single‐breath determination of carbon monoxide uptake in the lung. The European Respiratory Journal, 26, 720–735. 16204605 10.1183/09031936.05.00034905

[phy216184-bib-0037] McConnell, A. K. , & Copestake, A. J. (1999). Maximum static respiratory pressures in healthy elderly men and women: Issues of reproducibility and interpretation. Respiration, 66, 251–258. 10364742 10.1159/000029386

[phy216184-bib-0038] McElvaney, G. , Blackie, S. , Morrison, N. J. , Wilcox, P. G. , Fairbarn, M. S. , & Pardy, R. L. (1989). Maximal static respiratory pressures in the normal elderly. The American Review of Respiratory Disease, 139, 277–281. 2912349 10.1164/ajrccm/139.1.277

[phy216184-bib-0039] McNarry, M. A. , Berg, R. M. G. , Shelley, J. , Hudson, J. , Saynor, Z. L. , Duckers, J. , Lewis, K. , Davies, G. A. , & Mackintosh, K. A. (2022). Inspiratory muscle training enhances recovery post‐COVID‐19: A randomised controlled trial. The European Respiratory Journal, 60, 2103101. 35236727 10.1183/13993003.03101-2021 PMC8900538

[phy216184-bib-0040] Metcalfe, R. S. , Swinton, P. A. , Mackintosh, K. A. , Berg, R. M. G. , Shelley, J. , Saynor, Z. L. , Hudson, J. , Duckers, J. , Lewis, K. , Davies, G. A. , & McNarry, M. A. (2023). Heterogeneous treatment effects after inspiratory muscle training during recovery from Postacute COVID‐19 syndrome. Medicine and Science in Sports and Exercise, 55, 1761–1769. 37170947 10.1249/MSS.0000000000003207

[phy216184-bib-0041] Micro Direct Inc . (2019). Respiratory pressure meter operating manual.

[phy216184-bib-0042] Miller, M. R. , Hankinson, J. , Brusasco, V. , Burgos, F. , Casaburi, R. , Coates, A. , Crapo, R. , Enright, P. , van der Grinten, C. P. , Gustafsson, P. , Jensen, R. , Johnson, D. C. , MacIntyre, N. , McKay, R. , Navajas, D. , Pedersen, O. F. , Pellegrino, R. , Viegi, G. , & Wanger, J. (2005). Standardisation of spirometry. The European Respiratory Journal, 26, 319–338. 16055882 10.1183/09031936.05.00034805

[phy216184-bib-0043] Montes‐Ibarra, M. , Oliveira, C. L. P. , Orsso, C. E. , Landi, F. , Marzetti, E. , & Prado, C. M. (2022). The impact of long COVID‐19 on muscle health. Clinics in Geriatric Medicine, 38, 545–557. 35868672 10.1016/j.cger.2022.03.004 PMC8934728

[phy216184-bib-0044] Munkholm, M. , Marott, J. L. , Bjerre‐Kristensen, L. , Madsen, F. , Pedersen, O. F. , Lange, P. , Nordestgaard, B. G. , & Mortensen, J. (2018). Reference equations for pulmonary diffusing capacity of carbon monoxide and nitric oxide in adult Caucasians. The European Respiratory Journal, 52, 1500677. 29903858 10.1183/13993003.00677-2015

[phy216184-bib-0045] Nagel, C. , Olschewski, H. , Sorichter, S. , Uezgoer, G. , Diehm, C. , Huppert, P. , Iber, T. , Herth, F. , Harutyunova, S. , Marra, A. M. , Benjamin, N. , Salkić, A. , Grünig, E. , & Egenlauf, B. (2022). Impairment of inspiratory muscle function after COVID‐19. Respiration, 101, 981–989. 36310021 10.1159/000527361

[phy216184-bib-0046] Neder, J. A. , Andreoni, S. , Lerario, M. C. , & Nery, L. E. (1999). Reference values for lung function tests. II. Maximal respiratory pressures and voluntary ventilation. Brazilian Journal of Medical and Biological Research, 32, 719–727. 10412550 10.1590/s0100-879x1999000600007

[phy216184-bib-0047] Panel, C.‐T. G. (2022). Coronavirus Disease 2019 (COVID‐19) Treatment Guidelines. Accessed December 1, 2022. https://www.covid19treatmentguidelines.nih.gov/.

[phy216184-bib-0048] Plaza, M. , & Sevilla, G. G. P. (1992). Respiratory muscle sequelae in young university students infected by coronavirus disease 2019: An observational study. Revista Da Associacao Medica Brasileira, 68, 245–249. 10.1590/1806-9282.20211040 35239890

[phy216184-bib-0049] Ricoy, J. , Rodríguez‐Núñez, N. , Álvarez‐Dobaño, J. M. , Toubes, M. E. , Riveiro, V. , & Valdés, L. (2019). Diaphragmatic dysfunction. Pulmonology, 25, 223–235. 30509855 10.1016/j.pulmoe.2018.10.008

[phy216184-bib-0050] Rodrigues, A. , Da Silva, M. L. , Berton, D. C. , Cipriano, G., Jr. , Pitta, F. , O'Donnell, D. E. , & Neder, J. A. (2017). Maximal inspiratory pressure: Does the choice of reference values actually matter? Chest, 152, 32–39. 27940276 10.1016/j.chest.2016.11.045

[phy216184-bib-0051] Shi, Z. , de Vries, H. J. , Vlaar, A. P. J. , van der Hoeven, J. , Boon, R. A. , Heunks, L. M. A. , & Ottenheijm, C. A. C. (2021). Diaphragm pathology in critically ill patients with COVID‐19 and postmortem findings from 3 medical centers. JAMA Internal Medicine, 181, 122–124. 33196760 10.1001/jamainternmed.2020.6278 PMC7670391

[phy216184-bib-0052] Spiesshoefer, J. , Regmi, B. , Senol, M. , Jörn, B. , Gorol, O. , Elfeturi, M. , Walterspacher, S. , Giannoni, A. , Kahles, F. , Gloeckl, R. , & Dreher, M. (2024). Potential diaphragm muscle weakness‐related dyspnea persists two years after COVID‐19 and could be improved by inspiratory muscle training: Results of an observational and an interventional trial. American Journal of Respiratory and Critical Care Medicine. Epub ahead of print. 10.1164/rccm.202309-1572OC PMC11389583 38763165

[phy216184-bib-0053] Thomsen, M. , Nordestgaard, B. G. , Vestbo, J. , & Lange, P. (2013). Characteristics and outcomes of chronic obstructive pulmonary disease in never smokers in Denmark: A prospective population study. The Lancet Respiratory Medicine, 1, 543–550. 24461615 10.1016/S2213-2600(13)70137-1

[phy216184-bib-0054] Villelabeitia‐Jaureguizar, K. , Calvo‐Lobo, C. , Rodríguez‐Sanz, D. , Vicente‐Campos, D. , Castro‐Portal, J. A. , López‐Cañadas, M. , Becerro‐de‐Bengoa‐Vallejo, R. , & Chicharro, J. L. (2022). Low intensity respiratory muscle training in COVID‐19 patients after invasive mechanical ventilation: A retrospective case‐series study. Biomedicine, 10(11), 2807. 10.3390/biomedicines10112807 PMC9687222 36359327

[phy216184-bib-0055] Vincken, W. , Ghezzo, H. , & Cosio, M. G. (1987). Maximal static respiratory pressures in adults: Normal values and their relationship to determinants of respiratory function. Bulletin Européen de Physiopathologie Respiratoire, 23, 435–439. 3450325

[phy216184-bib-0056] Wanger, J. , Clausen, J. L. , Coates, A. , Pedersen, O. F. , Brusasco, V. , Burgos, F. , Casaburi, R. , Crapo, R. , Enright, P. , van der Grinten, C. P. , Gustafsson, P. , Hankinson, J. , Jensen, R. , Johnson, D. , Macintyre, N. , McKay, R. , Miller, M. R. , Navajas, D. , Pellegrino, R. , & Viegi, G. (2005). Standardisation of the measurement of lung volumes. The European Respiratory Journal, 26, 511–522. 16135736 10.1183/09031936.05.00035005

[phy216184-bib-0057] WHO . (2023). WHO Coronavirus (COVID‐19) Dashboard World Health Organization (WHO). Accessed December 11, 2023. https://covid19.who.int/.

[phy216184-bib-0058] Wilson, S. H. , Cooke, N. T. , Edwards, R. H. , & Spiro, S. G. (1984). Predicted normal values for maximal respiratory pressures in caucasian adults and children. Thorax, 39, 535–538. 6463933 10.1136/thx.39.7.535 PMC459855

[phy216184-bib-0059] Windisch, W. , Hennings, E. , Sorichter, S. , Hamm, H. , & Criée, C. P. (2004). Peak or plateau maximal inspiratory mouth pressure: Which is best? The European Respiratory Journal, 23, 708–713. 15176684 10.1183/09031936.04.00136104

[phy216184-bib-0060] Wohlgemuth, M. , van der Kooi, E. L. , Hendriks, J. C. , Padberg, G. W. , & Folgering, H. T. (2003). Face mask spirometry and respiratory pressures in normal subjects. The European Respiratory Journal, 22, 1001–1006. 14680093 10.1183/09031936.03.00028103

