# Supplementary material for: Maximal respiratory pressure after COVID‐19 compared with reference material in healthy adults: A prospective cohort study (The SECURe study)
Source: Physiol Rep. 2024 Sep 8;12(17):e16184. doi: 10.14814/phy2.16184 (PMC11381190; doi:10.14814/phy2.16184)
Supplement: Supplementary file 1 — Figure S1: [file PHY2-12-e16184-s004.docx]

**Supplementary figure 1**: Age distribution of the reference study population

**Supplementary figure 2**: The correlation of maximal inspiratory pressure and maximal expiratory pressure in female and males in the reference study. The line indicates the predicted maximal inspiratory pressure from maximal expiratory pressure using fractional polynomials.

**Supplementary figure 3**: The correlation of maximal inspiratory pressure and BMI in female and males in the reference study. The line indicates the predicted maximal inspiratory pressure from BMI using fractional polynomials.

**Supplementary figure 4**: The correlation of maximal expiratory pressure and BMI in female and males in the reference study. The line indicates the predicted maximal expiratory pressure from BMI using fractional polynomials.

**Supplementary figure 5**: The correlation of maximal inspiratory pressure and height in female and males in the reference study. The line indicates the predicted maximal inspiratory pressure from height using fractional polynomials.

**Supplementary figure 6**: The correlation of maximal expiratory pressure and height in female and males in the reference study. The line indicates the predicted maximal expiratory pressure from height using fractional polynomials.

**Supplementary figure 7:** The correlation of maximal inspiratory pressure and weight in female and males in the reference study. The line indicates the predicted maximal inspiratory pressure from weight using fractional polynomials.

**Supplementary figure 8:** The correlation of maximal expiratory pressure and weight in female and males in the reference study. The line indicates the predicted maximal expiratory pressure from weight using fractional polynomials.

**Supplementary figure 9**: The correlation of maximal inspiratory pressure and finger reach in female and males in the reference study. The line indicates the predicted maximal inspiratory pressure from finger reach using fractional polynomials.

**Supplementary figure 10**: The correlation of maximal expiratory pressure and finger reach in female and males in the reference study. The line indicates the predicted maximal expiratory pressure from finger reach using fractional polynomials.
