# Supplementary material for: Maximal respiratory pressure after COVID‐19 compared with reference material in healthy adults: A prospective cohort study (The SECURe study)
Source: Physiol Rep. 2024 Sep 8;12(17):e16184. doi: 10.14814/phy2.16184 (PMC11381190; doi:10.14814/phy2.16184)
Supplement: Supplementary file 2 — Table S1. [file PHY2-12-e16184-s001.docx]

**Supplementary table 1:** Multivariable linear regression of age, BMI, height or finger reach, and weight as correlates for maximal inspiratory pressure in 298 adults

|  | Adjusted for age and age^2^ | | Adjusted for age, age^2^, height or height and weight | | Adjusted for age, age^2^, finger reach or finger reach and weight | | Final model | |
| --- | --- | --- | --- | --- | --- | --- | --- | --- |
|  | B (95% CI) | p-value | B (95% CI) | p-value | B (95% CI) | p-value | B (95% CI) | p-value |
| **Male** |  |  |  |  |  |  |  |  |
| Age, years | 0.9 (-0.3;2.1) | 0.14 | 0.5 (-0.7;1.6) | 0.45 | 0.4 (-0.8; 1.7) | 0.47 | - - |  |
| Age^2^, years^2^ | -0.02 (-0.03;-0.004) | 0.007 | -0.01 (-0.02;0.001) | 0.078 | -0.01 (-0.02; -0.001) | 0.068 | -0.007 (-0.008; -0.005) | <0.001 |
| Height, cm |  |  | 0.5 (-0.3;1.2) | 0.23 | - - |  | - - |  |
| Finger reach, cm |  |  |  |  | 0.1 (-0.5; 0.7) | 0.70 |  |  |
| Weight, kg |  |  | 0.4 (-0.01;0.8) | 0.057 | 0.5 (0.1; 0.8) | 0.016 | 0.524 (0.198; 0.851) | 0.002 |
| Adjusted R^2^ | 0.26 | | 0.30 | | 0.29 | | 0.30 | |
| Residual standard error | 28.014 | | 27.286 | | 27.411 | | 27.285 | |
|  |  |  |  |  |  |  |  |  |
| **Female** |  |  |  |  |  |  |  |  |
| Age, years | 0.4 (-0.4;1.3) | 0.33 | 0.3 (-0.6;1.1) | 0.57 | 0.3 (-0.6; 1.1) | 0.52 | - - |  |
| Age^2^, years^2^ | -0.01 (-0.02;-0.002) | 0.014 | -0.01 (-0.02;0.001) | 0.074 | -0.01 (-0.02; -0.00003) | 0.049 | -0.005 (-0.007; -0.003) | <0.001 |
| Height, cm |  |  | 0.6 (0.03;1.1) | 0.039 | - - |  | 0.602 (0.070; 1.134) | 0.027 |
| Finger reach, cm |  |  |  |  | 0.6 (0.1; 1.1) | 0.024 | - - |  |
| Adjusted R^2^ | 0.31 | | 0.33 | | 0.33 | | 0.33 | |
| Residual standard error | 22.341 | | 22.094 | | 22.031 | | 22.045 | |
