# Supplementary material for: Maximal respiratory pressure after COVID‐19 compared with reference material in healthy adults: A prospective cohort study (The SECURe study)
Source: Physiol Rep. 2024 Sep 8;12(17):e16184. doi: 10.14814/phy2.16184 (PMC11381190; doi:10.14814/phy2.16184)
Supplement: Supplementary file 3 — Table S2. [file PHY2-12-e16184-s002.docx]

**Supplementary table 2:** Multivariable linear regression of age, BMI, height or finger reach, and weight as correlates for maximal expiratory pressure in 298 adults

|  | Adjusted for age and age^2^ | | Adjusted for age, age^2^, height or height and weight | | Adjusted for age, age^2^, finger reach or finger reach and weight | | Final model | |
| --- | --- | --- | --- | --- | --- | --- | --- | --- |
|  | B (95% CI) | p-value | B (95% CI) | p-value | B (95% CI) | p-value | B (95% CI) | p-value |
| **Male** |  |  |  |  |  |  |  |  |
| Age, years | 2.3 (0.8;3.8) | 0.003 | 1.9 (0.4;3.4) | 0.016 | 1.8 (0.3; 3.4) | 0.019 | 1.866 (0.344; 3.388) | 0.017 |
| Age^2^, years^2^ | -0.03 (-0.04;-0.02) | <0.001 | -0.02 (-0.04;-0.01) | 0.001 | -0.02 (-0.04; -0.01) | 0.001 | -0.025 (-0.039; -0.011) | <0.001 |
| Height, cm |  |  | 0.6 (-0.3;1.5) | 0.21 |  |  |  |  |
| Finger reach, cm |  |  |  |  | 0.3 (-0.4; 1.1) | 0.36 |  |  |
| Weight, kg |  |  | 0.3 (-0.1;0.8) | 0.17 | 0.4 (-0.1; 0.9) | 0.092 | 0.491 (0.067; 0.916) | 0.024 |
| Adjusted R^2^ | 0.30 | | 0.32 | | 0.32 | | 0.34 | |
| Residual standard error | 34.821 | | 34.244 | | 34.338 | | 34.318 | |
|  |  |  |  |  |  |  |  |  |
| **Female** |  |  |  |  |  |  |  |  |
| Age, years | 1.3 (0.3;2.4) | 0.012 | 1.2 (0.2;2.3) | 0.021 | 1.2 (0.2; 2.3) | 0.021 | 1.329 (0.292; 2.366) | 0.012 |
| Age^2^, years^2^ | -0.02 (-0.03;-0.01) | <0.001 | -0.02 (-0.03;-0.01) | 0.001 | -0.02 (-0.03; 0.01) | <0.001 | -0.018 (-0.027; -0.009) | <0.001 |
| Height, cm |  |  | 0.3 (-0.4;0.9) | 0.42 | - - |  |  | 0.027 |
| Finger reach, cm |  |  |  |  | 0.4 (-0.2; 1.0) | 0.21 |  |  |
| Adjusted R^2^ | 0.30 | | 0.30 | | 0.30 | | 0.30 | |
| Residual standard error | 26.831 | | 26.861 | | 26.781 | | 26.831 | |
