# Supplementary material for: Maximal respiratory pressure after COVID‐19 compared with reference material in healthy adults: A prospective cohort study (The SECURe study)
Source: Physiol Rep. 2024 Sep 8;12(17):e16184. doi: 10.14814/phy2.16184 (PMC11381190; doi:10.14814/phy2.16184)
Supplement: Supplementary file 4 — Table S3. [file PHY2-12-e16184-s007.docx]

**Supplementary table 3**: Reference equation using finger reach instead of height for maximal inspiratory pressure in females

| Maximal inspiratory pressure | Multiple linear regression equation | R squared | Residual standard error |
| --- | --- | --- | --- |
| Female | -1.093-0.0053∙age^2^+0.5878∙finger reach | 0.33 | 21.988 |
| Age in years and finger reach in cm  Maximal inspiratory and expiratory pressure in cmH_2_O | | | |
