# Supplementary material for: Maximal respiratory pressure after COVID‐19 compared with reference material in healthy adults: A prospective cohort study (The SECURe study)
Source: Physiol Rep. 2024 Sep 8;12(17):e16184. doi: 10.14814/phy2.16184 (PMC11381190; doi:10.14814/phy2.16184)
Supplement: Supplementary file 5 — Table S4. [file PHY2-12-e16184-s003.docx]

**Supplementary table 4:** Univariate linear regression of age, age^2^, BMI, height, finger reach, and weight as correlates for maximal inspiratory pressure in 267 adults with BMI ≤30

|  | **Female** | |  | **Male** | |  |
| --- | --- | --- | --- | --- | --- | --- |
|  | **B (95% CI)** | **p-value** | **R squared** | **B (95% CI)** | **p-value** | **R squared** |
| Age (years) | -0.6 (-0.8;-0.5) | <0.001 | 0.30 | -0.7 (-1.0;-0.5) | <0.001 | 0.22 |
| Age^2^ (years^2^) | -0.006 (-0.007;-0.004) | <0.001 | 0.32 | -0.007 (-0.009;-0.005) | <0.001 | 0.25 |
| Height (cm) | 1.3 (0.7;1.8) | <0.001 | 0.13 | 1.7 (1.0;2.3) | <0.001 | 0.16 |
| Weight (kg) | 0.4 (-0.05;0.9) | 0.082 | 0.02 | 1.4 (0.9;1.9) | <0.001 | 0.19 |
| BMI (kg/m^2^) | -0.7 (-1.7;0.3) | 0.18 | 0.01 | 3.0 (0.9;5.2) | 0.007 | 0.05 |
| Finger reach (cm) | 1.0 (0.4; 1.6) | 0.001 | 0.08 | 0.9 (0.3; 1.5) | 0.005 | 0.05 |
