# Supplementary material for: Maximal respiratory pressure after COVID‐19 compared with reference material in healthy adults: A prospective cohort study (The SECURe study)
Source: Physiol Rep. 2024 Sep 8;12(17):e16184. doi: 10.14814/phy2.16184 (PMC11381190; doi:10.14814/phy2.16184)
Supplement: Supplementary file 6 — Table S5. [file PHY2-12-e16184-s005.docx]

**Supplementary table 5:** Univariate linear regression of age, age^2^, BMI, height, finger reach, and weight as correlates for maximal expiratory pressure in 267 adults with BMI ≤30

|  | **Female** | |  | **Male** | |  |
| --- | --- | --- | --- | --- | --- | --- |
|  | **B (95% CI)** | **p-value** | **R squared** | **B (95% CI)** | **p-value** | **R squared** |
| Age (years) | -0.7 (-0.9;-0.5) | <0.001 | 0.22 | -0.9 (-1.2;-0.6) | <0.001 | 0.22 |
| Age^2^ (years^2^) | -0.006 (-0.008;-0.004) | <0.001 | 0.27 | -0.009 (-0.01;-0.006) | <0.001 | 0.27 |
| Height (cm) | 1.3 (0.6;2.0) | <0.001 | 0.09 | 2.2 (1.4;3.0) | <0.001 | 0.17 |
| Weight (kg) | 0.7 (0.2;1.3) | 0.04 | 0.01 | 1.2 (0.6;1.9) | <0.001 | 0.09 |
| BMI (kg/m^2^) | 0.3 (-1.8;2.3) | 0.78 | -0.007 | 1.0 (-1.8;3.8) | 0.50 | -0.004 |
| Finger reach (cm) | 1.0 (0.3; 1.7) | 0.006 | 0.05 | 1.2 (0.5; 2.0) | 0.002 | 0.06 |
