# Supplementary material for: Maximal respiratory pressure after COVID‐19 compared with reference material in healthy adults: A prospective cohort study (The SECURe study)
Source: Physiol Rep. 2024 Sep 8;12(17):e16184. doi: 10.14814/phy2.16184 (PMC11381190; doi:10.14814/phy2.16184)
Supplement: Supplementary file 7 — Table S6. [file PHY2-12-e16184-s006.docx]

**Supplementary table 6**: Reference equations for maximal inspiratory and expiratory pressure for persons with BMI ≤30

|  | **Multiple linear regression equation** | **R squared** | **Root mean squared error** |
| --- | --- | --- | --- |
| Maximal inspiratory pressure |  |  |  |
| Male | 37.0258-0.0061∙age^2^+1.0969∙weight | 0.37 | 26.232 |
| Female | 28.244-0.0052∙age^2^+0.4064∙height | 0.33 | 21.691 |
| Maximal expiratory pressure |  |  |  |
| Male | 74.647+1.7713∙age-0.0241∙age^2^+0.6735∙weight | 0.33 | 33.954 |
| Female | 73.375+1.1624∙age-0.0165∙age^2^+0.4345∙weight | 0.30 | 27.075 |
| Age in years, height in cm, and weight in kg  Maximal inspiratory and expiratory pressure in cmH_2_O | | | |
